# Supplementary figures and images for: The 2 Sigma Genus Concept in mammalogy: Lessons from Lasiurus
Source: PLoS One. 2025 Jun 25;20(6):e0325554. doi: 10.1371/journal.pone.0325554 (PMC12193745; doi:10.1371/journal.pone.0325554)

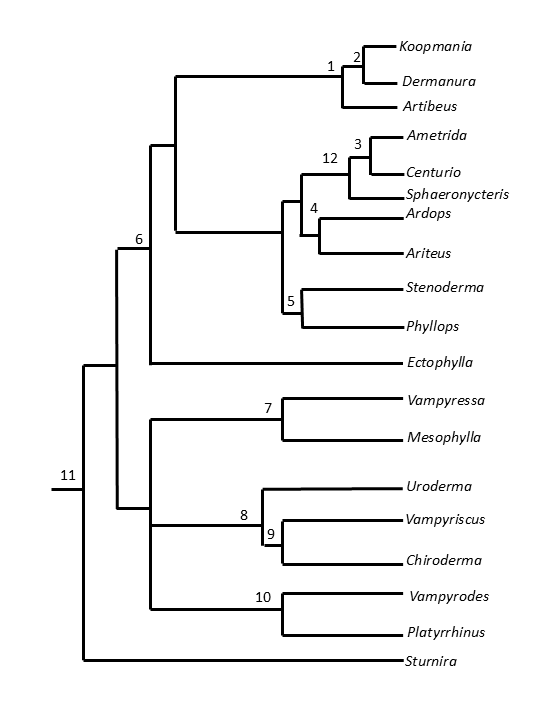

Supplement: S5 Fig — See text for discussion on relationships among Artibeus, Dermanura, and Koopmania. Numbers above nodes correspond to sister genera relationships analyzed. Distances among these sister genera are given in Supplementary S2 Table. (TIF) [file pone.0325554.s005.tif]
